# *PCSK3* Overexpression in Sjögren’s Syndrome Patients May Be Regulated by rs4932178 SNP in Its Promoter Region and Correlates with *IFN-γ* Gene Expression

**DOI:** 10.3390/genes14050981

**Published:** 2023-04-26

**Authors:** Andrea Latini, Giada De Benedittis, Serena Colafrancesco, Carlo Perricone, Giuseppe Novelli, Lucia Novelli, Roberta Priori, Cinzia Ciccacci, Paola Borgiani

**Affiliations:** 1 Department of Biomedicine and Prevention, Genetics Section, University of Rome Tor Vergata, 00133 Rome, Italy; a.latini@med.uniroma2.it (A.L.); dbngdi01@uniroma2.it (G.D.B.); novelli@med.uniroma2.it (G.N.); borgiani@med.uniroma2.it (P.B.); 2 Division of Rheumatology, Department of Clinical Internal, Anaesthesiologic and Cardiovascular Sciences, Sapienza University, 00133 Rome, Italy; serena.colafrancesco18@gmail.com; 3 Rheumatology, Department of Medicine, University of Perugia, Piazzale Giorgio Menghini 1, 06129 Perugia, Italy; carlo.perricone@gmail.com; 4 IRCCS NEUROMED, 86077 Pozzilli, Italy; 5 School of Medicine, Department of Pharmacology, University of Nevada, Reno, NV 89557, USA; 6 UniCamillus, Saint Camillus International University of Health Sciences, 00131 Rome, Italy; lnovelli@hotmail.it (L.N.); roberta.priori63@gmail.com (R.P.); 7 AOU Policlinico Umberto 1, 00161 Rome, Italy

**Keywords:** Sjögren’s syndrome, *PCSK3*, *IFN-γ*

## Abstract

Background: The *PCSK3* gene encodes for the protease enzyme Furin, which promotes proteolytic maturation of important regulators of the immune response, and also enhances the secretion of interferon-γ (IFN). Several studies have suggested its possible involvement in the pathogenesis of chronic inflammatory diseases. Methods: We investigated the *PCSK3* gene expression level in peripheral blood mononuclear cells isolated from Sjögren’s Syndrome (SS) patients and healthy controls and we evaluated a possible correlation with *IFN-γ* gene expression. Moreover, we also explored the variability of two *PCSK3* genetic polymorphisms (rs4932178 and rs4702) to evaluate a possible association between these polymorphisms and the expression levels of this gene. Results: We observed, by RT-qPCR, that the *PCSK3* expression level was significantly higher in SS patients compared to the controls (*p* = 0.028), and we confirmed a positive correlation between *PCSK3* and *IFN-γ* expression levels (*p* < 0.001). Moreover, we reported that the variant homozygous genotype of rs4932178 SNP is associated with a higher expression of the *PCSK3* gene (*p* = 0.038) and with the SS susceptibility (*p* = 0.016). Conclusions: Our data suggest that Furin could play a role in SS development, also promoting IFN-γ secretion.

## 1. Introduction

Sjögren’s Syndrome (SS; OMIM 270150) is a chronic inflammatory autoimmune disease. It is characterized by chronic lymphocytic infiltrates in the exocrine glands, resulting in dry eye and dry mouth. The spectrum of this disease may also include systemic manifestations such as arthritis, interstitial lung involvement, and neurological involvement [1]. Interactions between environmental stimuli and genetic susceptibility factors are involved in disease development [2], but SS etiology is still partially unknown. The identification of new genes associated with this disease could therefore help to gain a more complete view on the mechanisms involved in SS development.

Several studies have suggested the involvement of the *PCSK3* gene in the pathogenesis of chronic inflammatory diseases, such as atherosclerosis, rheumatoid arthritis (RA), and systemic lupus erythematous (SLE) [3,4,5]. The *PCSK3* gene, consisting of sixteen exons, is located on chromosome 15q26.1 and encodes for a convertase subtilisin/kexin enzyme (Furin) that promotes proteolytic maturation of pro-proteins, thanks to its cleavage properties [6]. Furin is an important regulator of the immune response, and it is known to exert a pro-inflammatory function [3]. Indeed, among its substrates, some have immunoregulatory functions such as cytokines, integrins, and proteins of the viral envelope [7]. However, Cordova et al. have shown that Furin expression could also reduce the production of pro-inflammatory cytokines in myeloid cells [8]. In addition, it is demonstrated that the expression of Furin in T cells is critical for the maintenance of peripheral immune tolerance [9]; in fact, its deletion in mouse T cells results in a loss of tolerance [10]. As in all autoimmune inflammatory conditions, tolerance defects also contribute to SS pathogenesis [11]. An upregulation of Furin has been observed in SS, particularly in salivary gland biopsies and peripheral circulation [12,13]. Moreover, Furin is a ubiquitously expressed protein, preferentially in Th1 cells, and promotes the secretion of interferon-γ (IFN) by activated T cells [10,14]. IFN-γ is known to mediate Th1 cell functions, macrophage activation, and immunoglobulin class switching, and it has been widely associated with systemic autoimmunity [15]. Recent studies in both animal models and patients have demonstrated that IFN type II (IFN-γ) is involved in the pathogenesis of SS. Indeed, over half of SS patients exhibit an IFN signature and high levels of IFN-γ were found in serum, saliva, and Th cells from salivary gland biopsies of these patients [16,17,18]. Moreover, in SS patients, IFN-γ levels correlate with the disease activity score, and the presence of inflammatory infiltration is closely related to IFN-γ activity [18].

Genetic variants located in regulatory regions of the *PCSK3* gene could influence its transcript levels. Indeed, it is known that the *PCSK3* expression is regulated by three promoters that activate the transcription of three mRNA isoforms with a different 5′-untranslated region (UTR) [19]. The rs4932178 SNP (Single Nucleotide Polymorphism) in the most active promoter of this gene has been described as associated with a higher mRNA level in vitro. *PCSK3* expression levels are regulated also by 3′UTR, where several miRNAs binding sites are located. Several studies suggest that a SNP (rs4702) in this region causes a differential binding of miR-338-3p and could influence the gene expression [20,21].

Here, we aimed to investigate the *PCSK3* gene expression level in peripheral blood mononuclear cells (PBMCs) isolated from SS patients and healthy controls (CTRLs) and to evaluate a possible correlation with *IFN-γ* gene expression. Moreover, we explored the variability of two *PCSK3* genetic polymorphisms (rs4932178 and rs4702), located in regulatory regions, to evaluate a possible association between these polymorphisms and their expression levels.

## 2. Materials and Methods

SS patients were enrolled from the Sjögren’s Clinic of Sapienza University of Rome and diagnosed according to the 2016 ACR-EULAR Classification Criteria [22]. Study protocol included a complete physical examination and blood drawing. The clinical and laboratory data were collected in a standardized filled form including demographics, past medical history, date of diagnosis, comorbidities, and previous/concomitant treatments. Regarding laboratory data, the following has been performed for each patient: a complete cell blood count, including the leucocyte, erythrocyte, and platelet counts; serum protein electrophoresis to determine levels of C3 and C4 complements, hypergammaglobulinemia, and monoclonal components; immunofluorescence (IIF) on Hep-2 to reveal antinuclear antibodies (ANA) (considered present at a titer 1:160); Enzyme-Linked Immunosorbent Assay (ELISA) to detect anti-SSA and anti-SSB antibodies; Waaler–Rose test and/or Ra test to detect the rheumatoid factor presence; cryoprecipitate detection to determine the cryoglobulins (samples kept at 37 °C, warm centrifugation, warm cell precipitation, serum conservation at 4 °C, and cryoprecipitate detection after 7 days).

Healthy controls were enrolled at the University of Rome “Tor Vergata”. Written informed consent was obtained from each participant and the ethical committee of Sapienza University of Rome approved the study design. Peripheral blood samples from all patients and controls have been collected and stored at −20 °C until usage. The expression study was performed in a group of 27 SS patients and 18 healthy controls. Demographic and clinical features of both groups were reported in Table 1.

Total RNA was isolated from PBMCs using the TRIzol reagent (Ambion, CA, USA) protocol, followed by reverse transcription using the High-Capacity cDNA Reverse Transcription Kit (Applied Biosystems, Waltham, MA, USA). Expression analysis was performed by quantitative RT-polymerase chain reaction (RT-qPCR) (SYBR Green Assay, Applied Biosystems), using the 7500 Real-Time PCR System (Applied Biosystems). *PCSK3* expression analysis was performed on SS patients and healthy controls, while *IFN-γ* mRNA levels was evaluated only on the SS group. The primers used to detect gene expression are: 5′-AAGATGACCCAGATCATGTTTGAGACC and 3′-AGCCAGGTCCAGACGCAGGAT for *β-Actin*; 5′-CGGAAAGTGAGCCACTCATA and 3′-TGTCTTTGGGCTCGGTGAG for *PCSK3*; 5′-GCATCCAAAAGAGTGTGGAG and 3′-GACAGTTCAGCCATCACTTGG for *IFN-γ*. Each sample was analysed in triplicate and, to standardize the results, each assay was run with an endogenous control (β-Actin). Relative expression levels were calculated using the 2^−ΔΔCt^ method and data were reported as mean values ± standard deviation.

Genomic DNA was isolated from PBMCs of 195 SS patients using a Qiagen blood DNA mini-kit. A demographic and clinical description of patients was previously reported [23]. Patients were analysed for two polymorphisms in the *PCSK3* gene (rs4932178 and rs4702), and genotyping was performed by direct sequencing (ABI 3130xl Automated Sequencer (Applied Biosystems, Foster City, CA, USA)) using the following primers: 5′-CATAATTGTGGCAGCACTGG and 3′-AGCACCTGGGATTCATCCTG for *PCSK3 rs4932178*; 5′-TTCCTGGTACCCAGCCATCT and 3′-CAGGCAGGCCACTGTGTAG for *PCSK3 rs4702*.

DNA and RNA quality and concentration were evaluated by the NanoDrop ND-1000 Spectrophotometer (Thermo Fisher Scientific, Waltham, MA, USA) and RNA integrity was assessed by standard denaturing agarose gel electrophoresis.

Pearson’s χ^2^ test has been used to verify the Hardy–Weinberg equilibrium for SNPs and to compare genotype frequencies of patients with respect to those listed in the 1000 Genomes Project database for the European non-Finnish population. Odds ratios with a 95% CI were calculated. The ANOVA test was used to compare expression values among the different phenotypic and genotypic groups. The linear regression analysis was used to evaluate the correlation between *PCSK3* and *IFN-γ* mRNA levels. A *p*-value < 0.05 was considered as significant.

All graphs were performed by GraphPad Prism 9 (GraphPad Software, Boston, MA, USA). All statistical analyses were performed by the SPSS program, version 25 (IBM Corp, Armonk, NY, USA).

## 3. Results

For this study, we included 27 patients with SS (88.5% women), with a mean age of 58.79 ± 10.33 years and a disease duration of 4.64 ± 5.01 years. Among the patients, 82.1% developed ANA, 64.3% anti-SSA, and 53.6% anti-SSB antibodies. Additionally, 14.3% of cases presented arthritis, 7.1% lymphoproliferative complications (non-Hodgkin lymphoma), and 3.6 salivary gland swelling. For the control group, we enrolled 18 sex- and age-matched healthy subjects (83.3% women; mean age of 59.3 ± 10.21 years) (Table 1).

**Figure 1:**
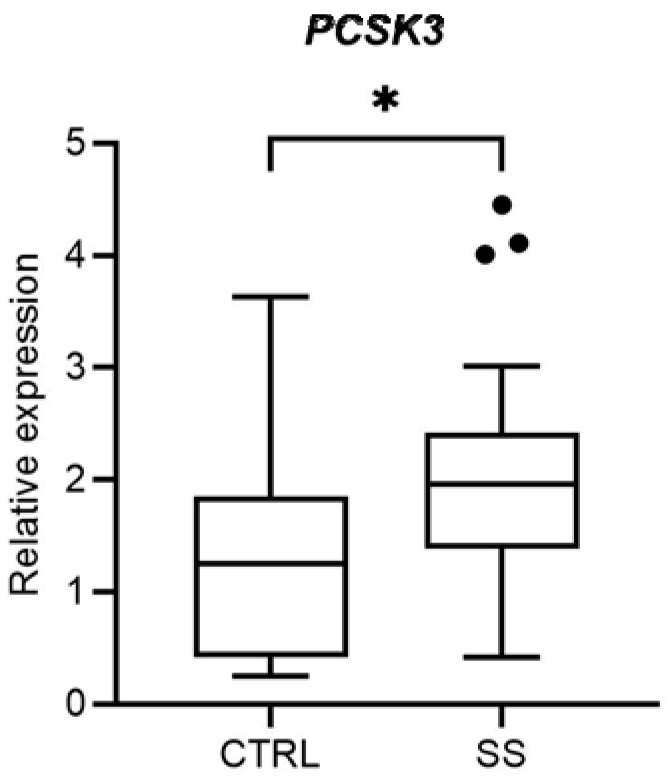
Comparison of *PCSK3* expression levels between healthy controls (CTRL) and Sjögren Syndrome patients (SS). * *p* = 0.028.

Firstly, we analysed the *PCSK3* gene expression in PBMCs of 27 SS patients and 18 healthy controls. We observed, by RT-qPCR, that *PCSK3* expression level was significantly higher in SS patients, compared to the controls (*p* = 0.028; Figure 1).

We also examined the possible correlation between *PCSK3* expression levels and specific clinical features of SS patients reported in Table 1, but we did not find any significant association.

**Figure 2:**
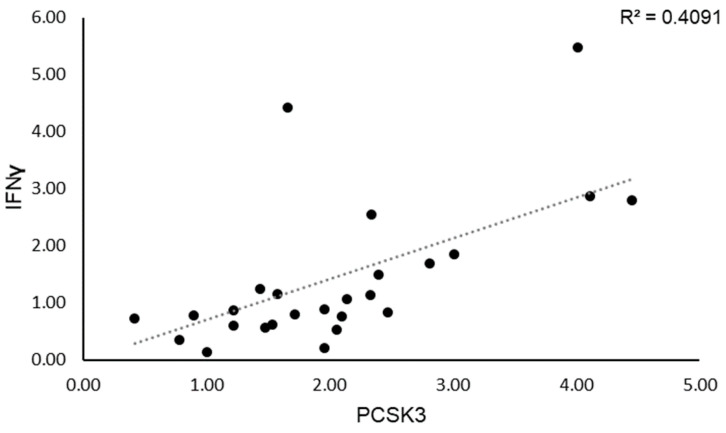
Correlation between *IFN-γ* and *PCSK3* expression levels.

We then evaluated whether *PCSK3* expression affects *IFN-γ* regulation in SS patients. We observed that SS patients with high *PCSK3* mRNA levels showed also elevated mRNA levels of *IFN-γ*; indeed, we confirmed a positive correlation between *PCSK3* and *IFN-γ* expression levels (R^2^ = 0.41; *p* < 0.001; Figure 2).

**Figure 3:**
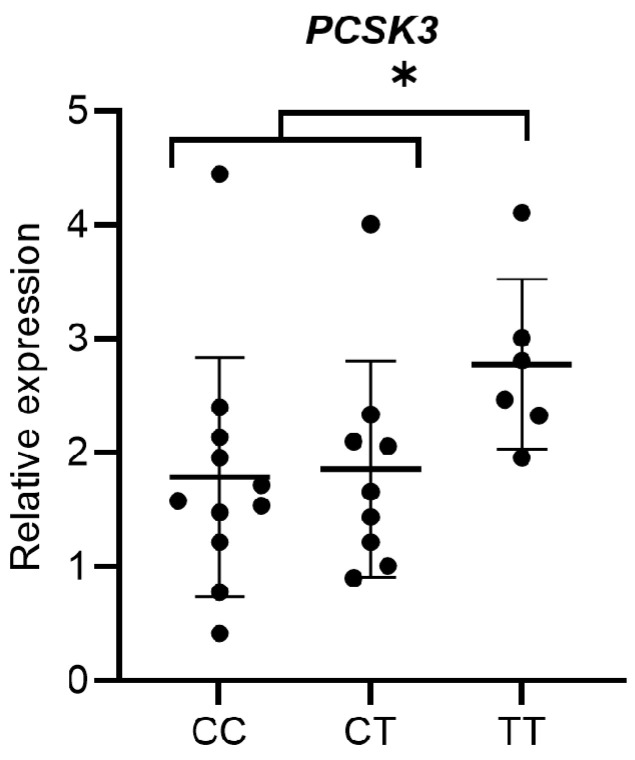
Distribution of mean expression levels of *PCSK3* among the genotypic classes for the polymorphism rs4932178. * *p* = 0.038.

To verify the possible association between common polymorphisms localized in the regulatory regions of the *PCSK3* gene and the different expression levels of the same gene, we compared the distribution of the mean values of *PCSK3* expression in the different genotypic classes for two selected SNPs in the SS patients. The analysed SNPs were rs4932178 and rs4702. As shown in Figure 3, the rs4932178 polymorphism variant allele in the *PCSK3* promoter region was associated with a higher expression of this gene. In particular, the patients with homozygous variant genotype (TT) showed an increase of *PCSK3* expression compared with the patients with homozygous wild-type (CC) and heterozygous (CT) genotypes (*p* = 0.038). No significant association was observed between the rs4702 polymorphism located in 3′-UTR region and the expression levels of *PCSK3* gene.

Lastly, we investigated the genotypes distribution of the two polymorphisms previously evaluated, in a cohort of 195 SS patients. We compared the distributions of genotypic frequencies of the two *PCSK3* polymorphisms (rs4932178 and rs4702) in the SS patients to the frequencies listed in the 1000 Genomes Project database for the European non-Finnish population. In the light of the higher expression level seen in the homozygous TT genotype, the associations were evaluated under a recessive genetic model. The *PCSK3* genotype distribution and the case/control association analysis are reported in Table 2.

As shown, the variant homozygous genotype (TT) of rs4932178 SNP was associated with SS susceptibility (*p* = 0.016 and OR = 1.71). Conversely, no statistically significant associations were observed for rs4702. To complete the association analysis, we also evaluated the recessive genetic model, but in this case, we did not observe any significant differences.

## 4. Discussion

Increasing evidence supports the role of the Furin enzyme in the regulation of peripheral immune tolerance by processing many substrates with immunoregulatory functions, including cytokines and integrins [7]. *PCSK3* expression is indeed upregulated in activated T cells and previous studies have shown that it is highly expressed in patients with autoimmune diseases, such as SLE [5] or RA [24].

In this study, in agreement with data previously reported by Ranta et al. [13], we observed an increase of *PCSK3* levels in PBMC of SS patients. Functional studies have demonstrated that *PCSK3* is indispensable in maintaining peripheral tolerance, and alteration of its expression levels could affect Treg cell function [10]. Indeed, inhibition of *PCSK3* was found to reduce the expression and activation of cytokines and growth factors, such as TGFβ [10]. We also reported a positive correlation between *PCSK3* and *IFN-γ* expression levels. The main pathophysiological mechanisms involved in SS are mediated through the interferon IFN I and IFN II pathways. In particular, in vivo studies have shown that reduction of IFN-γ seems to inhibit the development of SS [25]. Another study showed that *PCSK3* is one of the genes most consistently activated by IL-12, the main mediator of T-Helpers [26]. The authors also demonstrated that the secretion of IFN-γ was enhanced by Furin expression, while the inhibition of this enzyme interfered with IFN-γ production. However, it is not yet clear how this regulation takes place. Indeed, the authors had initially identified a potential Furin-target amino acid sequence on IFN-γ and hypothesized that the enzyme could directly cleave IFN-γ to promote its maturation. The introduction of amino acid mutations in this IFN-γ sequence did not alter the Furin enhance activity, suggesting a different form of regulation [26].

In the present work, we also described an association between rs4932178 SNP located in the *PCSK3* promoter region and the expression level of this gene. Specifically, patients with the homozygous variant genotype showed an increase of *PCSK3* expression and this genotype turned out to be also associated with a higher risk of developing SS. The *PCSK3* gene is located at position 15q26.1, and its transcription is mediated by three distinct promoters. Rs4932178 SNP is located in the most active promoter region, at −229 nucleotides from the start codon AUG. Lei et al. demonstrated using a luciferase reporter gene assay that transcription activity in variant allele carriers is about three times higher than in wild-type allele carriers of this SNP [19]. Indeed, they showed that the promoter with variant allele binds more efficiently the transcription factor NF-E2 [nuclear factor (erythroid-derived 2)] and induces a higher transcription activity of PCSK3 gene.

Moreover, we observed a different genotypic distribution of rs4932178 polymorphism in SS patients than reported in the 1000 Genomes Project database for the European non-Finnish population. In particular, the variant homozygous genotype (TT) seems to be more frequent in the SS cohort compared to the general population. Since this genotype appears to be associated with increased expression levels of PCSK3, it is not surprising that it is more frequent in SS patients, who, in fact, express higher levels of this enzyme.

During the last two years, Furin activity has also been extensively evaluated with regard to SARS-CoV-2 infection. Indeed, several studies demonstrated that the spike (S) protein of this virus contains a functional Furin-cleavage sequence (RRAR), essential for the membrane-fusion process and for the viral entry into the cell [27]. Moreover, genetic variants in the *PCSK3* gene have been described as possibly associated with SARS-CoV-2 infection susceptibility [28]. COVID-19 shares clinical manifestations and pathogenic mechanisms with autoimmune diseases and several immune reactions participate in the pathogenesis of both conditions [29,30]. Indeed, numerous immunity genes were found associated with COVID-19 susceptibility and severity [31,32]. Our study now suggests that *PCSK3*, already described as associated to COVID19, could also be included in the IFN-γ network and considered a novel susceptibility gene for autoimmune disorders.

## 5. Conclusions

In conclusion, we observed an increase of *PCSK3* mRNA levels in SS patients compared to control subjects and an association of variant homozygous genotype (TT) of rs4932178 SNP, both with a higher expression of this gene and with the SS susceptibility. Moreover, we observed a positive correlation between *PCSK3* and *IFN-γ* expression levels. This study has some limitations: the absence of data regarding the measurement of Furin protein levels by ELISA and the small size of the analysed cohort. Although replication studies on a larger cohort will be necessary to confirm our data, these results suggest for the first time that Furin could play a role in SS development. Functional studies are necessary to clarify the contribution of Furin to the SS pathogenesis.

## Figures and Tables

**Table 1:** Demographic and clinical features of SS patients and healthy controls.

	SS Patients (*n* = 27)	Healthy Control (*n* = 18)
Sex (% of famales)	88.5	83.3
Age (mean ± SD)	58.79 ± 10.33	59.3 ± 10.21
Age at diagnosis (mean ± SD)	54.14 ± 9.76	
Disease duration (means ± SD)	4.64 ± 5.01	
Xerophthalmia (%)	96.4	
Xerostomia (%)	85.7	
Salivary gland swelling (%)	3.6	
Arthritis (%)	14.3	
Lymphoma (%)	7.1	
ANA (%)	82.1	
Anti-SSA (%)	64.3	
Anti-SSB (%)	53.6	
Hypergammaglobulinemia (%)	39.3	
Rheumatoid factor (%)	23.1	
Leukopenia (%)	28.6	
Hypocomplementemia (%)	14.3	
Monoclonal component (%)	14.8	
Cryoglobulins (%)	0	

Quantitative data are expressed as mean and standard deviation (SD); Dichotomous data are expressed as a percentage. ANA were considered positive if titer ≥ 1:160; anti-SSA, anti-SSB, and RF were considered positive according to the cut-off of the reference laboratory; hypergammaglobulinemia was diagnosed if total Ig ≥ 20% of total proteins; leukopenia was diagnosed if WBC < 4000/mm^3^; hypocomplementemia was diagnosed if C3 < 80 mg/dL and/or C4 < 15 mg/dL.

**Table 2:** Association analysis between *PCSK3* SNPs and Sjögren Syndrome.

*PCSK3* rs4932178				Recessive Model (CC + CT vs. TT)	
	CC	CT	TT	*p*	OR (95%CI)
Sjögren Syndrome	78 (40.4%)	77 (39.9%)	38 (19.7%)	**0.016**	1.71 (1.10−2.66)
European 1000 Genomes project	208 (41.4%)	232 (46.1%)	63 (12.5%)		
***PCSK3* rs4702**				**Recessive Model (AG +GG vs. AA)**	
	**AA**	**AG**	**GG**	***p***	**OR (95%CI)**
Sjögren Syndrome	56 (28.7%)	97 (49.7%)	42 (21.5%)	0.75	1.06 (0.71−1.60)
European 1000 Genomes project	166 (33%)	234 (46.5%)	103 (20.5%)		

Significant associations are reported in bold.

## Data Availability

All available data may be found in the manuscript.
